# Dietary Supplement Use in Children and Adolescents Aged ≤19 Years — United States, 2017–2018

**DOI:** 10.15585/mmwr.mm6943a1

**Published:** 2020-10-30

**Authors:** Bryan Stierman, Suruchi Mishra, Jaime J. Gahche, Nancy Potischman, Craig M. Hales

**Affiliations:** ^1^Division of Health and Nutrition Examination Surveys, National Center for Health Statistics, CDC; ^2^Epidemic Intelligence Service, CDC; ^3^Office of Dietary Supplements, National Institutes of Health.

Dietary supplement use is common among children and adolescents. During 2013–2014, approximately one third of children and adolescents (persons aged ≤19 years) in the United States were reported to use a dietary supplement in the past 30 days, and use varied by demographic characteristics (*1*,*2*). Dietary supplements can contribute substantially to overall nutrient intake, having the potential to both mitigate nutrient shortfalls as well as to lead to nutrient intake above recommended upper limits (*3*). However, because nutritional needs should generally be met through food consumption according to the 2015–2020 Dietary Guidelines for Americans, only a few dietary supplements are specifically recommended for use among children and adolescents and only under particular conditions (*4*). The most recently released data from the National Health and Nutrition Examination Survey (NHANES) during 2017–2018 were used to estimate the prevalence of use among U.S. children and adolescents of any dietary supplement, two or more dietary supplements, and specific dietary supplement product types. Trends were calculated for dietary supplement use from 2009–2010 to 2017–2018. During 2017–2018, 34.0% of children and adolescents used any dietary supplement in the past 30 days, with no significant change since 2009–2010. Use of two or more dietary supplements increased from 4.3% during 2009–2010 to 7.1% during 2017–2018. Multivitamin-mineral products were used by 23.8% of children and adolescents, making these the products most commonly used. Because dietary supplement use is common, surveillance of dietary supplement use, combined with nutrient intake from diet, will remain an important component of monitoring nutritional intake in children and adolescents to inform clinical practice and dietary recommendations.

NHANES is a cross-sectional survey designed to monitor the health and nutrition of the civilian noninstitutionalized resident U.S. population (https://www.cdc.gov/nchs/nhanes/index.htm). The survey was approved by the National Center for Health Statistics Research Ethics Review Board. Signed consent from parents/guardians or participants aged ≥18 years, as well as documented assent from minor participants aged 7–17 years, were obtained. Information on dietary supplement use was obtained during an in-home interview. Participants aged ≥16 years and emancipated minors were interviewed directly. An adult proxy provided information for participants aged <16 years. Participants were asked to show product containers for all dietary supplements taken in the past 30 days. The interviewer recorded information from the product labels. The NHANES interview response rate for children and adolescents during 2017–2018 was 59.3%.

Age groups were categorized as <2, 2–5, 6–11, and 12–19 years. Self-reported race and Hispanic origin were divided into five categories: non-Hispanic White, non-Hispanic Black, non-Hispanic Asian, Hispanic, and “other.” The “other” category included non-Hispanic persons reporting other races or more than one race and was included in total estimates but not shown separately. Family income was categorized as ≤130%, >130% to ≤350%, and >350% of the federal poverty level (FPL) which accounts for inflation, family size, and geographic location. Highest educational attainment of the household head was divided into three categories: less than high school, high school graduate or equivalent or some college or an associate degree, and college graduate or above. All products were classified using mutually exclusive categories in the following order: 1) multivitamin-mineral products containing ≥3 vitamins and ≥1 mineral; 2) products containing primarily calcium with or without other ingredients; 3) products containing primarily omega-3 fatty acids with or without other ingredients; 4) products containing primarily probiotics with or without other ingredients; 5) products containing primarily fiber with or without other ingredients; 6) products containing primarily melatonin with or without other ingredients; 7) botanical products containing ≥1 botanical ingredient and no vitamins or minerals; 8) multivitamins containing ≥2 vitamins with no minerals; 9) amino acid products containing ≥1 amino acid; and 10) single nutrient supplements, categorized separately, such as single vitamins (e.g., vitamin D, vitamin C) and single minerals (e.g., iron). Results are presented for the product types most frequently used by children and adolescents, i.e., those with ≥1% prevalence of use.

Two participants with missing dietary supplement use data were excluded from analysis. All other 2017–2018 NHANES participants aged ≤19 years comprised the sample (n = 3,683). Analyses used 2-year interview weights and accounted for the survey’s complex, multistage probability design. Standard errors for proportions were calculated using Taylor series linearization, and 95% confidence intervals were constructed using the Korn and Graubard method (*5*). Reliability of estimates was assessed using the National Center for Health Statistics Data Presentation Standards for Proportions (*6*). Differences in dietary supplement use by sex, age group, and race and Hispanic origin were evaluated using pairwise comparisons with univariate two-sided t-statistics. Trends across income and education of household head were tested using linear regression including categories as continuous variables. Recent trends over the last 10 years from 2009–2010 to 2017–2018 were tested using orthogonal polynomial regression with 2-year NHANES cycles. Differences in product use by age group were tested using F-based second-order Rao-Scott tests. All reported differences are statistically significant (p<0.05). All analyses were performed using R (version 3.6.0; R Foundation for Statistical Computing), SAS (version 9.4; SAS Institute), and SUDAAN (version 11; RTI International).

During 2017–2018, overall prevalence of dietary supplement use among children and adolescents in the preceding 30 days was 34.0% (Table 1). Supplement use among females (37.3%) was higher than that among males (30.8%), and use prevalence was highest among those aged 2–5 years (43.3%) followed by those aged 6–11 years (37.5%), 12–19 years (29.7%), and <2 years (21.8%). Prevalence was higher among non-Hispanic Asian (41.1%) and non-Hispanic White children and adolescents (39.9%) compared with that among non-Hispanic Black (20.8%) and Hispanic (26.9%) children and adolescents. Dietary supplement use increased with increasing income and education of the head of household. Prevalence of use of two or more dietary supplements was 7.1% and varied by age, race and Hispanic origin, income, and education of the head of household.

**TABLE 1:** Prevalence of any dietary supplement use and use of two or more dietary supplements in the past 30 days among children and adolescents (persons aged ≤19 years), by selected characteristics — United States, 2017–2018

Characteristic	No.	% (95% CI)	
		Any dietary supplement	≥2 dietary supplements
**Total**	**3,683**	**34.0 (30.2–37.9)**	**7.1 (5.6–8.9)**
**Sex**			
Female	1,829	37.3 (33.7–41.0)	7.7 (5.9–9.8)
Male	1,854	30.8 (25.2–36.9)*	6.5 (4.5–9.2)
**Age group (yrs)**			
<2	591	21.8 (16.3–28.2)	2.4 (0.6–6.1)^†^
2–5	784	43.3 (37.6–49.2)^§^	8.3 (6.1–10.9)^§^
6–11	1,115	37.5 (33.5–41.6)^§,¶^	5.9 (3.6–8.9)
12–19	1,193	29.7 (24.1–35.7)^§,¶,^**	8.5 (5.9–11.6)^§^
**Race, Hispanic origin**			
White, non-Hispanic	1,214	39.9 (33.5–46.5)^††,§§^	8.6 (5.9–11.9)^††^
Black, non-Hispanic	816	20.8 (16.4–25.8)	1.8 (0.7–3.7)
Asian, non-Hispanic	357	41.1 (32.1–50.6)^††,§§^	9.3 (5.3–14.8)^††^
Hispanic	935	26.9 (20.9–33.6)	6.0 (3.6–9.2)^††^
**Family income relative to poverty level**			
≤130% of FPL	1,328	23.5 (16.6–31.7)	4.0 (1.9–7.2)^†^
>130% to ≤350% of FPL	1,209	34.5 (29.2–40.2)	7.0 (5.5–8.7)
>350% of FPL	706	45.9 (39.2–52.8)^¶¶^	11.1 (6.7–17.0)^¶¶^
**Education of household head**			
Less than high school graduation or equivalent	638	17.8 (11.9–25.0)	1.8 (0.6–4.2)
High school graduation or equivalent or some college or associate degree	2,030	33.7 (29.0–38.6)	6.9 (5.2–8.8)
College graduate or above	788	46.0 (39.3–52.9)***	10.8 (7.6–14.6)***

**Abbreviations:** CI = confidence interval; FPL = federal poverty level.

* Significantly different (p<0.05) from females.

^†^ Estimate does not meet standards of reliability.

^§^ Significantly different (p<0.05) from age group <2 years.

^¶^ Significantly different (p<0.05) from age group 2–5 years.

** Significantly different (p<0.05) from age group 6–11 years.

^††^ Significantly different (p<0.05) from non-Hispanic Black children and adolescents.

^§§^ Significantly different (p<0.05) from Hispanic children and adolescents.

^¶¶^ Statistically significant linear trend (p<0.05) for household income.

*** Statistically significant linear trend (p<0.05) for education of household head.

Among persons aged 12–19 years, use of any dietary supplement increased significantly in a linear fashion from 2009–2010 (22.1%) to 2017–2018 (29.7%) (Figure). Use of two or more dietary supplements increased significantly from 2009–2010 to 2017–2018 among all children and adolescents (from 4.3% to 7.1%) as well as among those aged 2–5 years (from 6.8% to 8.3%) and 12–19 years (from 3.2% to 8.5%).

**FIGURE:**
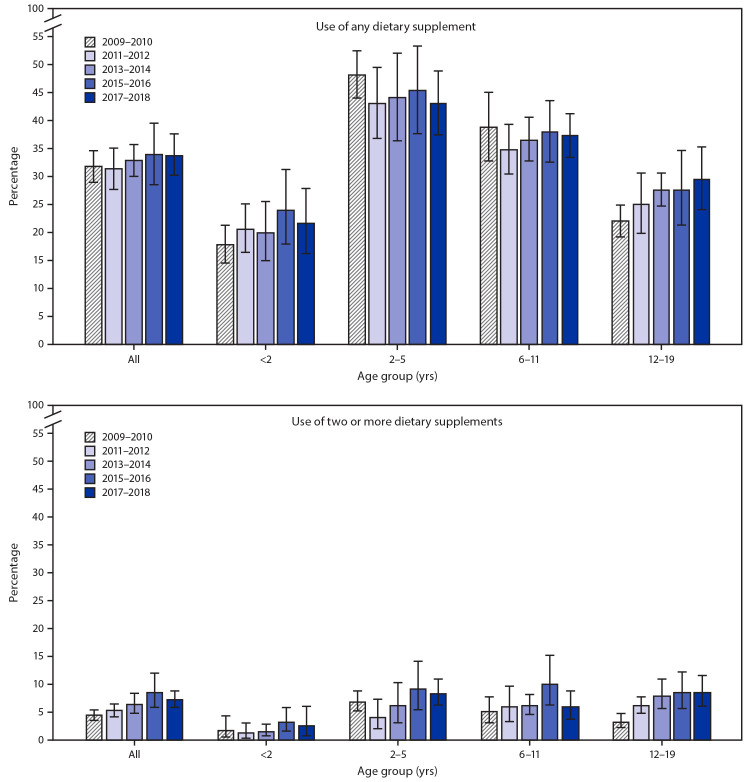
Prevalence of use of any dietary supplement* and use of two or more dietary supplements*^,^^†^ in the past 30 days among children and adolescents aged ≤19 years, by age group — United States, 2009–2010 to 2017–2018 * Statistically significant linear trend (p<0.05) for any dietary supplement use in age group 12–19 years and use of two or more dietary supplements in all ages and in age groups 2–5 and 12–19 years. ^†^ Estimate does not meet standards of reliability for use of two or more supplements for age group <2 years in 2017–2018 and 2–5 years in 2011–2012.

Multivitamin-minerals were the most used product type (23.8% of children and adolescents) (Table 2). Prevalence of use of single ingredient vitamin D (3.6%), single ingredient vitamin C (3.0%), probiotic (1.8%), melatonin (1.3%), omega-3 fatty acid (1.3%), botanical (1.1%), and multivitamin (1.0%) products all met or exceeded 1.0%. Multivitamin-mineral, single ingredient vitamin D, probiotic, and botanical product use differed by age group.

**TABLE 2:** Prevalence of use of most frequently used dietary supplement product types in the past 30 days among children and adolescents (persons aged ≤19 years), by age group — United States, 2017–2018

Product type	Age group (years), % (95% CI)					P-value for difference by age*
	All (n = 3,683)	<2 (n = 591)	2–5 (n = 784)	6–11 (n = 1,115)	12–19 (n = 1,193)	
Multivitamin-mineral	23.8 (20.3–27.7)	11.0 (7.3–15.5)	34.6 (28.8–40.7)	29.5 (24.5–34.8)	17.3 (13.7–21.4)	<0.001
Single ingredient vitamin D supplement	3.6 (2.2–5.5)	5.4 (2.8–9.3)	1.7 (0.6–3.9)	1.8 (0.8–3.5)	5.4 (2.9–9.0)	<0.001
Single ingredient vitamin C supplement	3.0 (1.9–4.4)	1.1 (0.1–4.6)	2.0 (0.7–4.4)	2.4 (1.6–3.5)	4.2 (2.1–7.6)	0.083
Probiotic	1.8 (1.1–2.8)	1.9 (0.7–4.3)	3.7 (1.9–6.2)	2.0 (0.7–4.6)	0.8 (0.3–1.7)	0.020
Melatonin	1.3 (0.7–2.2)	0.0 (0.0–0.6)	1.4 (0.5–3.0)	1.3 (0.7–2.3)	1.5 (0.5–3.2)	0.435
Omega-3 fatty acid	1.3 (0.6–2.4)	0.5 (0.1–1.6)	1.4 (0.5–3.0)	1.2 (0.3–3.1)	1.4 (0.3–3.9)	0.707
Botanical	1.1 (0.6–1.9)	0.5 (0.1–1.8)	0.5 (0.1–1.3)	0.3 (0.0–0.9)	2.1 (0.8–4.6)	0.001
Multivitamin	1.0 (0.5–1.6)	1.2 (0.3–3.2)	0.8 (0.2–2.0)	0.6 (0.1–1.6)	1.3 (0.6–2.4)	0.361

**Abbreviation:** CI = confidence interval.

* p-values calculated using F-based second-order Rao-Scott test.

## Discussion

During 2017–2018, approximately one third of children and adolescents used dietary supplements in the past 30 days. Prevalence among female children and adolescents exceeded that among males. Sex-differences in any dietary supplement use among all children and adolescents combined have not been reported previously; however, a 2013–2014 study found a large, non-significant difference in any dietary supplement use among adolescent females and males (*1*). As previously reported, dietary supplement use prevalence increased with income and education of household head (*2*). Patterns of dietary supplement use by age group and race and Hispanic origin also remained similar (*2*).

Use of two or more dietary supplements varied by age group, race and Hispanic origin, income, and education of household head and increased from 2009–2010 to 2017–2018. Few studies have examined use of multiple dietary supplements in children and adolescents. Dietary supplements may contain 100% or more of daily nutrient intake recommendations (*7*); therefore, use of two or more dietary supplements could lead to intakes above recommended upper limits if the products contain any of the same ingredients. Future studies could examine common combinations of dietary supplements used and their contribution to overall nutrient intake.

As with previous studies, multivitamin-minerals were the most frequently used dietary supplement products (*1*,*2*). Use of some product types varied by age. A recent study found that among U.S. children and adolescents who use dietary supplements, 18% took a dietary supplement under the recommendation of a health care provider (*2*). Few dietary supplement products are recommended for use among children and adolescents, and these are recommended only under specific circumstances. For example, the American Academy of Pediatrics (AAP) recommends that breastfeeding infants aged >4 months receive iron supplementation until introduction of iron-containing complementary foods and that all exclusively breastfed infants receive vitamin D supplementation (*8*,*9*). Other circumstances warranting dietary supplement use in children or adolescents include restrictive diets, pregnancy, and various illnesses. The 2015–2020 Dietary Guidelines for Americans recommend that nutritional needs be met primarily through food consumption; however, it recognizes that dietary supplements might be useful in some cases to compensate for nutrients that would otherwise be underconsumed (*4*). Dietary supplement use might mitigate nutrient shortfalls but might also lead to intake above recommended upper limits for some nutrients (*3*). AAP recommends that pediatric health care providers inquire about dietary supplement use among patients (*10*).

The findings in this report are subject to at least two limitations. First, the low prevalence of use of two or more dietary supplements make these estimates less reliable among some subgroups. Second, the lack of universal definitions for dietary supplement product types limits comparisons across studies.

Dietary supplement use is fairly prevalent among U.S. children and adolescents and contributes to overall total nutrient intake. NHANES will continue to provide information on dietary supplement use among children and adolescents to help inform clinical practice and policy, such as the Dietary Guidelines for Americans.

**Summary**

**What is already known about this topic?**

Approximately one third of U.S. children and adolescents take dietary supplements; use varies by demographic characteristics.

**What is added by this report?**

The most recently released dietary supplement use estimates from the 2017–2018 National Health and Nutrition Examination Survey (NHANES) demonstrate that dietary supplement use remained stable and prevalent among U.S. children and adolescents aged ≤19 years (34.0%). Use of two or more dietary supplements differed by demographic characteristics and increased from 2009–2010 (4.3%) to 2017–2018 (7.1%).

**What are the implications for public health practice?**

NHANES will continue to measure dietary supplement use among children and adolescents to inform clinical practice and dietary recommendations.

## References

[1] Qato DM, Alexander GC, Guadamuz JS, Lindau ST. Prevalence of dietary supplement use in US children and adolescents, 2003–2014. JAMA Pediatr 2018;172:780–2. 10.1001/jamapediatrics.2018.1008 29913013 PMC6142922

[2] Jun S, Cowan AE, Tooze JA, Dietary supplement use among U.S. children by family income, food security level, and nutrition assistance program participation status in 2011–2014. Nutrients 2018;10:1212. 10.3390/nu10091212 PMC6163871 30200511

[3] Bailey RL, Fulgoni VL 3rd, Keast DR, Lentino CV, Dwyer JT. Do dietary supplements improve micronutrient sufficiency in children and adolescents? J Pediatr 2012;161:837–42. 10.1016/j.jpeds.2012.05.009 22717218 PMC3477257

[4] US Department of Health and Human Services; US Department of Agriculture. 2015–2020 Dietary Guidelines for Americans. 8th ed. Washington, DC; 2015. https://health.gov/dietaryguidelines/2015/guidelines/

[5] Korn EL, Graubard BI. Confidence intervals for proportions with small expected number of positive counts estimated from survey data. Surv Methodol 1998;24:193–201.

[6] Parker JD, Talih M, Malec DJ, National Center for Health Statistics Data Presentation Standards for Proportions. Vital Health Stat 2 2017; (175):1–22. 30248016

[7] Bailey RL, Gahche JJ, Lentino CV, Dietary supplement use in the United States, 2003-2006. J Nutr 2011;141:261–6. 10.3945/jn.110.133025 21178089 PMC3021445

[8] Baker RD, Greer FR; Committee on Nutrition American Academy of Pediatrics. Diagnosis and prevention of iron deficiency and iron-deficiency anemia in infants and young children (0-3 years of age). Pediatrics 2010;126:1040–50. 10.1542/peds.2010-2576 20923825

[9] Wagner CL, Greer FR; American Academy of Pediatrics Section on Breastfeeding; American Academy of Pediatrics Committee on Nutrition. Prevention of rickets and vitamin D deficiency in infants, children, and adolescents. Pediatrics 2008;122:1142–52. 10.1542/peds.2008-1862 18977996

[10] McClafferty H, Vohra S, Bailey M, Pediatric integrative medicine. Pediatrics 2017;140:e20171961. 10.1542/peds.2017-1961 28847978

